# Sarcopenia, Precardial Adipose Tissue and High Tumor Volume as Outcome Predictors in Surgically Treated Pleural Mesothelioma

**DOI:** 10.3390/diagnostics12010099

**Published:** 2022-01-03

**Authors:** Oliver Guido Verhoek, Lisa Jungblut, Olivia Lauk, Christian Blüthgen, Isabelle Opitz, Thomas Frauenfelder, Katharina Martini

**Affiliations:** 1 Institute of Diagnostic and Interventional Radiology, University Hospital Zurich, Rämistrasse 100, 8091 Zurich, Switzerland; o.verhoek@gmail.com (O.G.V.); Lisa.Jungblut@usz.ch (L.J.); christian.bluethgen@usz.ch (C.B.); thomas.frauenfelder@usz.ch (T.F.); 2 Faculty of Medicine, University of Zurich, Rämistrasse 71, 8006 Zurich, Switzerland; Olivia.Lauk@usz.ch (O.L.); Isabelle.Schmitt-Opitz@usz.ch (I.O.); 3 Department of Thoracic Surgery, University Hospital Zurich, Rämistrasse 100, 8091 Zurich, Switzerland

**Keywords:** pleural mesothelioma, sarcopenia, tumor volume, adipose tissue, outcome

## Abstract

Background: We evaluated the prognostic value of Sarcopenia, low precardial adipose-tissue (PAT), and high tumor-volume in the outcome of surgically-treated pleural mesothelioma (PM). Methods: From 2005 to 2020, consecutive surgically-treated PM-patients having a pre-operative computed tomography (CT) scan were retrospectively included. Sarcopenia was assessed by CT-based parameters measured at the level of the fifth thoracic vertebra (TH5) by excluding fatty-infiltration based on CT-attenuation. The findings were stratified for gender, and a threshold of the 33rd percentile was set to define sarcopenia. Additionally, tumor volume as well as PAT were measured. The findings were correlated with progression-free survival and long-term mortality. Results: Two-hundred-seventy-eight PM-patients (252 male; 70.2 ± 9 years) were included. The mean progression-free survival was 18.6 ± 12.2 months, and the mean survival time was 23.3 ± 24 months. Progression was associated with chronic obstructive pulmonary disease (COPD) (*p* = <0.001), tumor-stage (*p* = 0.001), and type of surgery (*p* = 0.026). Three-year mortality was associated with higher patient age (*p* = 0.005), presence of COPD (*p* < 0.001), higher tumor-stage (*p* = 0.015), and higher tumor-volume (*p* < 0.001). Kaplan-Meier statistics showed that sarcopenic patients have a higher three-year mortality (*p* = 0.002). While there was a negative correlation of progression-free survival and mortality with tumor volume (r = 0.281, *p* = 0.001 and r = −0.240, *p* < 0.001; respectively), a correlation with PAT could only be shown for epithelioid PM (*p* = 0.040). Conclusions: Sarcopenia as well as tumor volume are associated with long-term mortality in surgically treated PM-patients. Further, while there was a negative correlation of progression-free survival and mortality with tumor volume, a correlation with PAT could only be shown for epithelioid PM.

## 1. Introduction

Pleural mesothelioma (PM) is a malignant and very aggressive cancer of the pleural surface [1]. The PM incidence has risen in the last ten years and predictions indicate that it will continue rising [2]. Patient survival in PM is very poor; even with the most advanced surgical techniques, median survival ranges only from 15 to 22 months [3,4]. Further, due to the radical and extensive nature of surgical techniques employed in PM, patients have a high post-operative morbidity and mortality [5,6]. Thus, thorough patient selection is of utmost importance to only select individuals with a positive predicted outcome and minimal risk of mortality for this extensive surgery.

There are already several clinico-pathological features to predict the outcome of surgery in patients with PM. These variables include age, weight loss, dyspnea, anemia, leukocytosis, thrombocytosis, tumor volume, C-reactive protein (CPR) level, epithelial tumor histology, and white blood cell count [7,8]. To more accurately predict the outcome of patients undergoing surgery in PM, the identification of further clinical and biological markers is the goal of ongoing research.

Cachexia comes with a host of complications and is estimated to be the main cause of death in up to 50% of cancer patients [9]. Cachexia is defined by loose of adipose tissue, lean body mass (LBM) and muscle tissue [10,11]. These parameters are quantifiable and a muscle-loss below a certain threshold is called sarcopenia. There have been several studies, including a large meta-analysis, which have shown a significant increase in mortality for cancer patients who are suffering from sarcopenia [12,13]. However, this correlation has not yet been investigated for patients suffering from PM.

Another aspect of cachexia, as described above, is the loss of adipose tissue. A relatively new but established method of measuring fatty tissue is to estimate the amount of mediastinal or precardial adipose tissue (PAT) in computer tomography scans [14,15]. There have been studies which showed that PAT can be used as pre-operative outcome predictors in some surgical treatments [16]. Similar correlations with post-operative outcome in PM-patients have not yet been showed.

Another key feature of predicting the outcome of any kind of oncological surgery is the volume of the tumor involved [17,18,19]. There have already been some studies which analyzed the effect of tumor volume as a prognostic marker for the surgical treatment of PM patients [20,21,22].

The purpose of this study was to evaluate if different CT derived morphometric measures, such as the 33rd percentile of muscle area at the fifth thoracic vertebra as a surrogate for sarcopenia, precradial adipose-tissue and high-tumor volume can be used to predict the outcome of surgically treated PM-patients. Furthermore, the aim of this analysis is to define selection criteria for surgical candidates in PM, which can indicate positive outcomes and minimal risk of postoperative mortality more accurately.

## 2. Materials and Methods

### 2.1. Patients

From September 2005 to November 2020, consecutive surgically-treated PM patients having a pre-operative computed tomography (CT) scan were retrospectively included. The study protocol was approved by the institutional review board and local ethics committee (StV 29-2009, EK-ZH 2012-0094; 07.03.2019 EK-ZH 2019-00369) and written informed consent was obtained from all patients. The work has been carried out in accordance with The Code of Ethics of the World Medical Association (Declaration of Helsinki).

### 2.2. Imaging

All included patients underwent routine preoperative CT on 16 to 64-detector CT units from different vendors at tube voltages of 100 to 140 kVp with or without contrast media injection. Images were reconstructed with a soft tissue convolution kernel at slice thicknesses from 0.75 to 3.0 mm.

### 2.3. Morphometric Measurements

#### 2.3.1. Muscle Area and Sarcopenia

**Figure 1:**
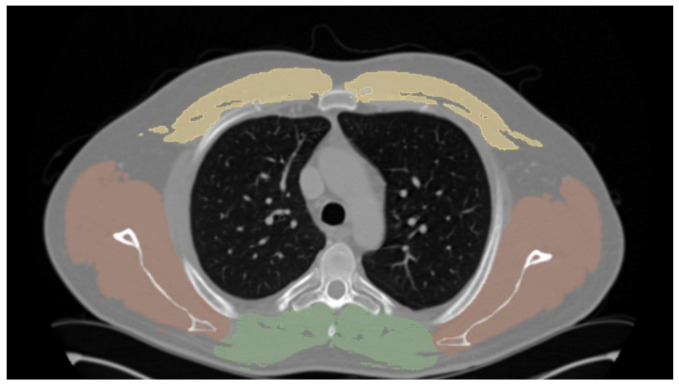
Morphometric measurements cross-sectional total paraspinal area (TPA, green), total rotator-cuff area (TRA, red), and total pectoral area (TPeA, yellow). Total muscle area (TMA) was defined as the sum of the former three measurements segmented on axial cross-sectional plane at the level of the fifth thoracic vertebra. Both transverse processes are visible in this plane.

Sarcopenia was semiautomatically assessed by CT-based parameters measured at the level of the fifth thoracic vertebra by excluding fatty-infiltration using a CT-attenuation threshold of −29 to 150 Hounsfield units (HU). According to the literature [23], sarcopenia was defined as less than the sex-matched 33rd percentile of the respective muscle area: cross-sectional total paraspinal area (TPA), total rotator-cuff area (TRA), and total pectoral area (TPeA). Total muscle area (TMA) was defined as the sum of the former three measurements (Figure 1).

#### 2.3.2. Mediastinal Fat

**Figure 2:**
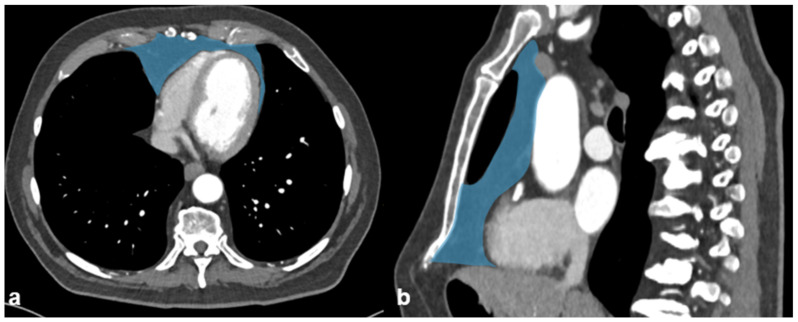
Morphometric measurements of anterior mediastinal fat on (**a**) axial and (**b**) sagittal reformations.

Mediastinal fat was semi-automatically assessed by outlining the mediastinal fat using a CT-attenuation threshold between −195 and −15 HU (Figure 2).

#### 2.3.3. Tumor Volume

**Figure 3:**
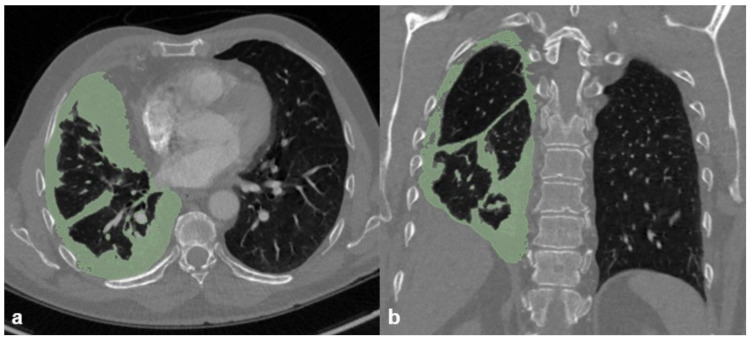
Morphometric measurements of tumor volume on (**a**) axial and (**b**) coronal reformations.

Tumor volume was assessed by outlining the tumor burden in the thoracic cage. Metastasis or lymph node metastasis were not measured (Figure 3).

### 2.4. Long-Term Outcome

As long-term outcome after surgically treated PM overall mortality and tumor progression were assessed. Progression was defined as tumor recurrence, tumor progression or death.

#### Statistical Analysis

Continuous variables were expressed as mean ± SD, and categorical variables were expressed as frequencies or percentages. Mann-Whitney and Kruskal-Wallis tests were used to perform group comparisons as appropriate. Further, survival analysis was performed using Kaplan-Meier statistics. Tumor volume as well as anterior mediastinal fat volume were correlated to tumor progression and survival using Pearson correlation (r) and linear logistic regression (R). All statistical analyses were conducted with the statistical software SPSS (SPSS, release 26.0; SPSS, Chicago, IL, USA).

## 3. Results

### 3.1. Patient Population

Overall, from September 2005 to November 2020 two-hundred-seventy-eight consecutive surgically-treated PM patients (252 male; 70.2 ± 9 years) having a pre-operative computed tomography (CT) scan were retrospectively included. Two-hundred-three patients (94%) additionally underwent neoadjuvant chemotherapy before undergoing surgical treatment. One-hundred-twenty patients (43.2%) underwent pleurectomy/decortication (P/D), thirty-seven (13.3%) partial pleurectomy, ninety-five (34.2%) extrapleural pneumonectomy (EPP), and twenty-five (9.2%) other types of surgery. Mean time interval from pre-operative CT to surgery was 20 days (SD 23). Overall, 161 patients (63%) had epithelioid PM, 9 patients (4%) sarcomatoid PM, 76 patients (30%) biphasic PM, and in 8 patients (3%) histologic type was not defined.

### 3.2. Morphometric Measurements

#### 3.2.1. Muscle Area and Sarcopenia

Mean total muscle area was of 18,661.9 ± 4517 mm^2^. Muscle area in males was significantly higher compared to their female counterparts (19,191.7 mm^2^ vs. 13,526.5 mm^2^; *p* = <0.001) (Table 1).

#### 3.2.2. Anterior Mediastinal Fat Volume

Mean anterior mediastinal fat volume was of 25.6 ± 15 cm^3^. Anterior mediastinal fat volume in males was significantly higher compared to their female counterparts (27.0 cm^3^ vs. 13.0 cm^3^; *p* = 0.001) (Table 1).

#### 3.2.3. Tumor Volume

Mean tumor volume was of 225.3 ± 268 mm^3^. Tumor volume in males was not significantly different from values of their female counterparts (228.6 cm^3^ vs. 198.5 cm^3^; *p* = 0.590) (Table 1). Tumor volume in male subjects with a history of asbestos exposure was significantly lower than tumor volume in male patients without a history of asbestos exposure (194.2 cm^3^ vs. 331.9 cm^3^; *p* = 0.035).

### 3.3. Outcome

Mean time to progression was of 18.6 months (SD 11.7). Mean survival time was of 23.3 months (SD 24).

#### 3.3.1. Progression Free Survival

Progression was associated with higher tumor stage (*p* = 0.001), presence of chronic obstructive pulmonary disease (*p* = 0.015) in patients where P/D was performed (*p* = 0.026), as well as PAT in epithelioid PM (*p* = 0.044). The different muscle surface values defining sarcopenia were not associated with tumor progression (Table 2).

Pearson correlation showed a negative correlation between tumor progression and tumor volume (r = −0.281, *p* < 0.001) as well as with PAT (r = −0.152, *p* < 0.037). While linear regression confirmed this correlation for tumor volume (R = 0.281, R^2^ 0.079, *p* < 0.001), the correlation between progression free survival and PAT could not be confirmed (R = 0.152, R^2^ 0.023, *p* = 0.073). However, when evaluating epithelioid PM separately, linear regression showed a correlation between progression free survival and PAT (epithelioid: R = 0.320, R^2^ 0.102, *p* = 0.004).

#### 3.3.2. Mortality

**Figure 4:**
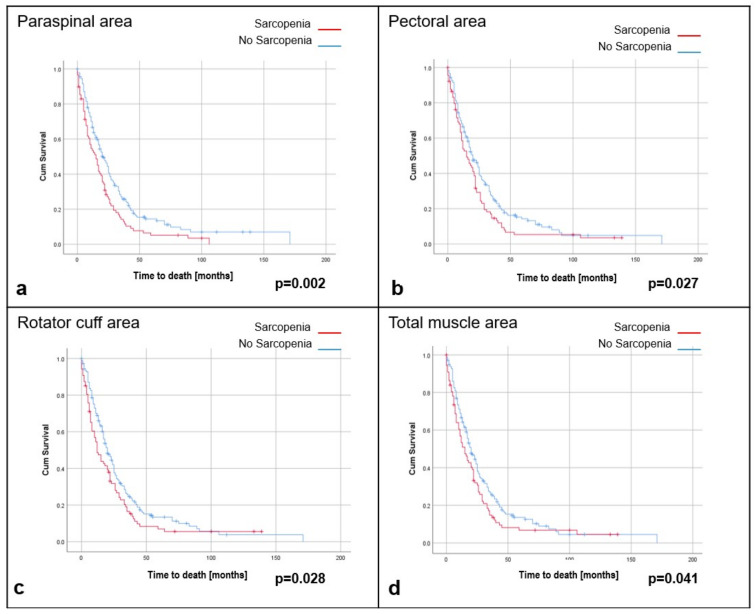
Kaplan-Meier statistics for surgically treated PM-patients with or without sarcopenia, defined as the sex-related 33rd percentile of the (**a**) paraspinal area, (**b**) pectoral area, (**c**) rotator cuff area, and (**d**) total muscle area at the 5th thoracic vertebra.

Three-year mortality was associated with higher patient age (*p* = 0.005), presence of COPD (*p* = <0.001), higher tumor stage (*p* = 0.015), and higher tumor volume (*p* < 0.001) (Table 2, Figure 4).

Kaplan-Meier statistics showed that sarcopenic patients have higher three-year mortality after surgically treated PM, with TPA performing best (*p* = 0.002) (Table 3).

While there was a negative correlation between mortality and tumor volume (r = −0.240, *p* < 0.001), correlation between mortality and PAT could not be shown (overall r = −0.066, *p* = 0.251; epithelioid r = 0.208, *p* = 0.0.64; biphasic r = −0.102, *p* = 0.594). Linear regression showed a correlation between mortality and tumor volume (R = 0.240, R^2^ 0.058, *p* < 0.001). Overall, linear regression did not show any correlation between mortality and PAT (R = 0.066, R^2^ 0.004, *p* = 0.503). However, when evaluating epithelioid and biphasic PM separately, linear regression showed a correlation between mortality and PAT (epithelioid: R = 0.063, R^2^ 0.004, *p* < 0.001; biphasic: R = 0.018, R^2^ 0.000, *p* < 0.001).

## 4. Discussion

The understanding of body composition in relation to chronic illnesses has been the focus of several studies in the last years. Sarcopenia, high tumor-volume, and low precardial adipose-tissue were reported to be associated with poor post-operative outcome in cancer-patients [13,14,15,21,24,25] In this study, we hypothesized that outcome in PM patients is affected by these factors in a similar way and could show that sarcopenia defined as the sex-related 33rd percentile of TPA at the level of the fifth thoracic vertebra as well as tumor volume are associated with long-term mortality in surgically treated PM patients. While there was a negative correlation of progression-free survival and mortality with tumor volume, correlation with PAT could only be shown for epithelioid PM.

There have been several studies who have shown that sarcopenia has a strong connection with overall survival in cancer patients [26,27,28,29,30] and patients with chronic disease [30,31,32,33,34,35,36]. Martini et al., evaluated the short-term outcome after pneumonectomy in lung cancer patients and found that sarcopenia defined as the gender-related 33rd percentile of fat-excluded total muscle area at the level of the third lumbar vertebra was associated with higher incidence of respiratory failure, ARDS and 30-day mortality [27]. Hsu and Kao et al. [35] analyzed the impact of sarcopenia on patients with chronic liver disease and concluded that Sarcopenia was not only a predictor for survival of patients on the waitlist of liver transplants, but also correlated with the pre-/and post-transplant adverse outcomes. Otten et al. [12], who evaluated the value of sarcopenia in different cancer patients, showed that the presence of sarcopenia correlated very strongly as a predictor of 1-year mortality and correlated nearly as strongly as advanced tumor stage.

In line with these studies, to the best of our knowledge we were the first to show that sarcopenia is associated with shorter three-year survival in surgically treated PM-patients. While tumor progression was associated with higher tumor stage, presence of chronic obstructive pulmonary disease, and type of operation, progression was not associated with the different muscle surface values defining sarcopenia.

At the moment, there is no consensus on the definition criteria of sarcopenia [37,38]. Traditionally, walking speed or general musculoskeletal activity were used to assess frailty of patients [39,40]. These measures, however, are very difficult if not impossible to objectify. In an attempt to make the evaluation more precise, different other approaches have been evaluated in the last years: Hasselager at al. [38] quantified sarcopenia by evaluating tricipital skin fold thickness and brachial circumference and could show that these measurements predicted poor long-term survival of patients suffering from surgically treated non-small cell lung cancer. Ruby et al. [41] used speed of sound ultrasound as a quantitative indicator for muscle loss and fatty muscular degeneration in seniors. A widely used and established approach to determine sarcopenia is the quantification of muscle tissue on cross sectional images [25,26,27,28]. Some authors propose to perform measurements at the third lumbar vertebra, others at the thoracic level [28,38,42,43,44,45,46]. Additionally, there are some studies to give evidence, that the heights of measurements do not play a major role in the definition: Nemec et al. [43] compared muscle mass at the level of TH12 and TH7 and found very strong correlation with the muscle mass measured at L3. Swartz et al. [44] showed that muscle mass measurements at the level of the third cervical vertebra correlate very strongly with the one measured at the level of the third lumbar vertebra. In our study, we measured the muscle mass according to Fintelmann et al. [47] at the thoracic level at height of the fifth thoracic vertebra (TH5). This was motivated by practical reasons, since chest CT is part of the routine workup in all PM-patients and therefore all PM patients from our department could be included.

The main interest in recognizing sarcopenia as risk-factor resides on its potential reversibility. Rehabilitation programs, physical therapy and proper nutrition can potentially reverse sarcopenia and have a favorable impact on surgical outcome. Further studies are needed to show if in patients where sarcopenia could be reversed with preoperative rehabilitations programs have better outcome as those who were not.

Mediastinal adipose tissue was shown to be a potential predictor for the frailty of patients [14,15,48] and there have been studies which showed that PAT can be used as pre-operative outcome predictor in some surgical treatments [16]. In our study overall, we did not find evidence to support this claim in PM patients. Correlation of progression-free survival and mortality with PAT could only be shown in a subgroup of patients, namely for epithelioid PM. One aspect of this is that it is very difficult to accurately measure PAT in patients with PM since the tumor is often located in close proximity to the mediastinum or infiltration of mediastinal fat is present. These factors make a differentiation of tumor mass from mediastinal fatty tissue potentially difficult and measurements prone to segmentation errors. With a significantly lager patient population this measurement errors could possibly be minimized and there could have potentially been significant results.

Interestingly, while asbestos exposure correlated with worse outcome in terms of death and progression free survival, male patients having asbestos exposure in their history showed to have lower tumor volume compared to their male counterparts without asbestos exposure.

As already shown in different malignancies [17,18,49], it was not surprising that in our study cohort tumor volume also correlated well with time to progression and survival. This goes in line with Pass et al. [50], who showed that tumor volume is a very good and accurate predictor for overall and progression-free survival in PM patients.

The limitations of this study are as follows. First, we measured muscle mass at TH5. Although, the most common assessment method to quantify skeletal muscle area are measurements at the level of the third lumbar vertebra, there have been studies as described above who have shown that measurements at different locations in the thoracoabdominal region have strong correlations [44, 44]. Second, tissue measurements were performed on contrast and non-contrast enhanced scans with the same HU thresholds. Even though this approach might lead to small measurement errors, previous studies have used the same technique and could show positive results [28, 28]. Third, slice thicknesses in CT images ranged from 0.75 to 3.0 mm. Different slice thicknesses result in different image noise levels and have different impact on partial volume which can result in measurement errors. The software we used for semiautomatic volume measurements takes into account different slice thicknesses in volume calculation, and since the threshold to defined sarcopenia was set on the base of the evaluated cohort, the measurement error plays a minor role in our study. Fourth, measurements were only performed by one reader. Due to the semiautomated process of deriving data with the help of sophisticated computer software, we would expect similar results for additional observers. Fifth, the retrospective nature of the study leading to an inhomogeneous patient cohort undergoing different therapy approaches. Sixth, we did not distinguish if patients underwent surgery with a curative or a palliative intent. This will have a negative impact on overall survival time and time to progression. However, the impact of PAT and sarcopenia will not be affected.

In conclusion, our study shows that tumor volume and sarcopenia are predictive markers for patient outcome in surgically treated PM. While there was a negative correlation of progression-free survival and mortality with tumor volume, a correlation with PAT could only be shown for epithelioid PM.

## Figures and Tables

**Table 1:** Morphometric measurements.

	Overall (Mean ± SD)	Male (Mean ± SD)	Female (Mean ± SD)	*p*-Value
Muscle area (mm^2^)				
Pectoral area at TH5	3759.1 ± 1141	3883.6 ± 1105	2552.1 ± 711	<0.001
Rotator cuff area at TH5	10,699.7 ± 2503	10,996.8 ± 2382	7820.4 ± 1709	<0.001
Paraspinal area at TH5	4203.1 ± 1854	4311.3 ± 1903	3154.1 ± 670	0.002
Total muscle area at TH5	18,661.9 ± 4517	19,191.7 ± 4324	13,526.5 ± 2849	<0.001
Precardial fat volume (cm^3^)	25.6 ± 15	27.0 ± 15	13.0 ± 9	0.001
Tumor volume (mm^3^)	225.3 ± 268	228.6 ± 272	198.5 ± 242	0.590
Patients with Asbestos exposure	194.2 ± 194	194.2 ± 194	*	
Patients without Asbestos exposure	286.9 ± 287	331.9 ± 307	200.9 ± 242	0.095

TH5 Level of the fifth thoracic vertebra, SD Standard deviation. * no female subject with asbestos exposure.

**Table 2:** Patient characteristics—post operative outcome.

	Total n = 250 *	3-Year Death n = 199 (72.6%)	3-Year Alive n = 51 (18.3%)	*p*-Value	Total n = 249 *	3-Year Progress n = 241 (96.8%)	No 3-Year Progress n = 8 (3.2%)	*p* Value
Male gender, n (%)	226 (90)	183 (92)	43 (84)	0.099	21 (8)	18 (25)	2 (8)	0.087
Age (years), mean (±SD)	70.4 (9)	71.2 (8)	67.3 (11)	0.005	70.6 (9)	70.6 (9)	70.5 (9)	0.737
Asbestos exposure, n (%)	136 (54)	130 (65)	6 (12)	<0.001	136 (55)	134 (56)	2 (25)	<0.001
Chronic cardiac disease, n (%)	17 (7)	12 (6)	5 (10)	0.352	17 (7)	17 (7)	0 (0)	0.420
COPD, n (%)	13 (5)	5 (3)	8 (16)	<0.001	13 (5)	11 (5)	2 (25)	0.015
Diabetes mellitus, n (%)	23 (9)	18 (6)	5 (10)	0.887	21 (8)	20 (8)	1 (13)	0.727
Preoperative FEV1 (% predicted), mean (±SD)	80.7 (19)	78.7 (18)	87.9 (20)	0.005	80.2 (19)	79.8 (18)	91.3 (26)	0.081
FEV1/FVC, mean (±SD)	88.6 (16)	88.2 (17)	89.9 (15)	0.567	88.3 (16)	88.3 (16)	88.7 (19)	0.588
Neoadjuvant Chemotherapy, n (%)	203 (81)	126 (63)	41 (75)	0.125	203 (82)	198 (82)	5 (63)	0.440
Histologyc type								
epithelioid, n (%)	159 (74)	142 (71)	17 (33)	<0.001	159 (64)	152 (63)	7 (89)	0.157
sarcomatoid, n (%)	9 (4)	8 (4)	1 (2)	0.481	9 (4)	9 (4)	0 (0)	0.268
biphasic, n (%)	74 (34)	71 (36)	3 (6)	<0.001	74 (30)	73 (30)	1 (13)	0.279
not defined, n (%)	8 (4)	7 (4)	1 (2)	0.573	8 (3)	7 (3)	1 (13)	0.130
*IMIG* stage, n (%) n = 238				0.015				0.001
I & II	144 (58)	114 (57)	30 (59)		166 (67)	162 (67)	4 (49)	
III & IV	1 (1)	0 (0)	1 (2)		45 (18)	43 (18)	2 (25)	
Type of surgery								
P/D	103 (41)	82 (41)	21 (41)	0.919	101 (41)	101 (42)	1 (13)	0.026
Partial pleurectomy	31 (12)	23 (12)	8 (16)	0.396	34 (14)	33 (14)	1 (13)	0.964
EPP	92 (37)	74 (37)	18 (35)	0.877	90 (36)	86 (36)	4 (50)	0.241
Other	23 (9)	20 (10)	3 (6)	0.215	0 (0)	0 (0)	0 (0)	---
Sarcopenia (calculated on muscle surface (cm^2^)), mean (±SD)								
Pectoral area at TH5	3754.6 (1166)	3701.1 (1110)	3963.2 (1355)	0.152	3740.6 (1119)	3743.5 (1124)	3652.4 (995)	0.391
Rotator cuff area at TH5	10,678.1 (2545)	10,584 (2540)	11,043.5 (2558)	0.251	10,692.0 (2534)	10,695.5 (2558)	10,586.9 (1739)	0.338
Paraspinal area at TH5	4212.5 (1940)	4106.8 (1501)	4624.8 (3098)	0.089	4205.5 (1942)	4218.9 (1965)	3802.4 (1000)	0.739
Total muscle area at TH5	18,645.1 (4630)	18,392.3 (4450)	18,811.1 (3410)	0.088	18,638.1 (4587)	18,657.9 (4628)	18,041.7 (3272)	0.340
Precardial fat volume (cm^3^)	25.1 (15)	26.9 (16)	20.1 (10)	0.130	25.6 (15)	26.1 (16)	24.3 (14)	0.863
epithelioid PM	21.4 (8)	22.1 (8)	18.0 (6)	0.044	22.0 (22)	22.4 (7)	7.9 (13)	**
biphasic PM	23.4 (10)	23.5 (10)	23.6 (9)	0.989	23.5 (10)	23.5 (10)	30.5 (19)	0.656
Tumor volume (mm^3^)	232.3 (273)	265.6 (290)	109.2 (146)	<0.001	242.6 (275)	245.2 (278)	152.4 (150)	0.329
epithelioid PM	236.2 (310)	250.8 (263)	107.3 (187)	0.023	248.9 (312)	251.6 (315)	154.3 (18)	0.372
biphasic PM	237.6 (181)	237.8 (182)	219.3 (189)	0.540	236.2 (181)	236.4 (182)	312.0 (215)	0.624

Clinical continuous variables are reported as mean ± standard deviation (SD). Categorical variables are reported as number (percentages). COPD, chronic obstructive pulmonary disease; FEV, forced expiratory volume; FVC, forced vital capacity; IMIG stage, International Mesothelioma Interest Group (8th Edition). P/D, pleurectomy/decortication; EPP, extrapleural pneumonectomy. Sarcopenia was defined as less than the gender specific 33rd percentile of the respective muscle area, thoracic vertebra level 5 (TH5). Pleural mesothelioma (PM). * patients were excluded due to a follow-up period < 36 months. ** not calculated, less than five patients in one group.

**Table 3:** Kaplan Meier—Survival.

	No Sarcopenia	Sarcopenia	*p*-Value
	Mean 3-Year Survival [95% CI]	Mean 3-Year Survival [95% CI]	
Sarcopenia (calculated on Total muscle surface (cm^2^))			
Pectoral area at TH5	32.3 [26–39]	22.8 [17–29]	0.027
Rotator cuff area at TH5	31.7 [25–38]	23.6 [17–30]	0.028
Paraspinal area at TH5	34.5 [27–42]	20.4 [15–25]	0.002
Total muscle area at TH5	31.7 [25–38]	23.9 [25–35]	0.041

Confidence interval (CI), thoracic vertebra level 5 (TH5).

## Data Availability

The data presented in this study are available on request from the corresponding author. The data are not publicly available due to ethical reasons.
